# Acute visual loss after ipilimumab treatment for metastatic melanoma

**DOI:** 10.1186/s40425-016-0170-9

**Published:** 2016-10-18

**Authors:** Melissa A. Wilson, Kelly Guld, Steven Galetta, Ryan D. Walsh, Julia Kharlip, Madhura Tamhankar, Suzanne McGettigan, Lynn M. Schuchter, Leslie A. Fecher

**Affiliations:** 1 Division of Hematology/Oncology, Department of Medicine, University of Pennsylvania, Philadelphia, PA USA; 2 Department of Medicine, University of Pennsylvania, Philadelphia, PA USA; 3 Department of Neurology, NYU Langone Medical Center, New York, NY USA; 4 Departments of Ophthalmology and Neurology, Medical College of Wisconsin, Milwaukee, WI USA; 5 Division of Endocrinology, Department of Medicine, University of Pennsylvania, Philadelphia, PA USA; 6 Department of Ophthalmology, Scheie Eye Institute, University of Pennsylvania Health System, Philadelphia, PA USA; 7 Present address: Division of Hematology and Medical Oncology, Laura and Isaac Perlmutter Cancer Center, NYU Langone Medical Center, New York, NY USA; 8 Present address: Division of Hematology/Oncology, Department of Internal Medicine, University of Michigan, C366 MIB 1500 E. Medical Center Drive, SPC5848, Ann Arbor, MI 48109 USA; 9 Present address: Department of Cardiology, UCSF Medical Center, San Francisco, CA USA

**Keywords:** Melanoma, Ipilimumab, Immune, Side effects, Optic neuritis, Checkpoint inhibitors

## Abstract

**Background:**

Ipilimumab, a humanized CLTA-4 antibody is a standard therapy in the treatment of advanced melanoma. While ipilimumab provides an overall survival benefit to patients, it can be associated with immune related adverse events (IrAEs).

**Case presentation:**

Here we describe a patient treated with ipilimumab who experienced known IrAEs, including hypophysitis, as well as a profound vision loss due to optic neuritis. There are rare reports of optic neuritis occurring as an adverse event associated with ipilimumab treatment. Furthermore, the patient experienced multiple complications from high dose steroids used to manage his IrAEs.

**Conclusions:**

This case highlights the need for recognition of atypical immune mediated processes associated with newer checkpoint inhibitor therapies including ipilimumab.

## Background

Until recently, there were limited treatment options for patients with advanced stage melanoma. In 2011, the United States Food and Drug Association approved two new treatments for advanced, unresectable melanoma - the antagonist cytotoxic T-lymphocyte antigen 4 (CTLA-4) antibody, ipilimumab, and the targeted BRAF inhibitor, vemurafenib. These new medications have demonstrated significant benefits in overall survival [1–3]. Since then, additional therapies have been approved for the treatment of advanced stage melanoma, both targeted therapies [4–8] and immunotherapies, including the newly FDA approved antagonist programmed cell death protein 1 (PD-1) antibodies, pembrolizumab and nivolumab [9–12]. Immuno-oncology is a growing field in the treatment of not only melanoma, but also other solid tumors, such as non-small cell lung and renal cell cancer [13]. These new treatments demonstrate improved and durable responses, but have unique, immune-related side effects which require prompt recognition and management distinct from traditional cytotoxic chemotherapies.

Ipilimumab is a human monoclonal antibody directed against CTLA-4. In the normal immune system, CTLA-4 downregulates activation of T-cells in hosts by interaction with a co-receptor on the antigen presenting cell. Antagonism of CTLA-4 with ipilimumab therefore blocks co-receptor interaction leading to activation of the innate immune system, stimulating an immune response against melanoma tumor cells [14]. It is well understood that treatment with ipilimumab can result in a number of immune related adverse events (IrAEs), felt to be the result of cross-reactive tissue damage by activated T-cells [15, 16]. Most IrAEs generally respond and resolve with steroid treatment for several weeks or longer. However, there are cases of IrAEs refractory to steroids that require additional immunosuppressive interventions, including intravenous immunoglobulin (IVIG) and Infliximab [17–19]. The most common immune-related side effects from treatment with ipilimumab include: colitis - causing diarrhea, endocrinopathies - such as hypophysitis and hypothyroidism, hepatitis, and dermatitis [20–25].

Ophthalmologic complications from ipilimumab therapy are rare, occurring in less than 1 % of patients, but generally manifest as uveitis [26–28] with symptoms of blurred vision, decreased visual acuity, dry eyes, pain, and photophobia [28, 29]. An association of colitis with uveitis in patients has been reported [28]. Thyroid like orbitopathy [16, 30–33] and ocular inflammation involving choroid and retina [34–36] have also been reported. Optic neuritis, which involves inflammation of the optic nerve is associated with a number of conditions including multiple sclerosis and autoimmune disorders. Several biologic agents, (especially tumor necrosis factor (TNF)-alpha inhibitors like infliximab, etanercept, and adalimumab) have been associated with optic neuritis [15]. Optic neuritis was recently described in association with ipilimumab treatment [33, 37]. We report here a patient who demonstrated multiple immune-related adverse events including panhypopituitarism and optic neuritis, resulting in monocular blindness, after undergoing treatment with ipilimumab. Moreover, treatment of the immune-related side effects with high dose steroids induced a catatonic depression requiring electroconvulsive therapy (ECT).

## Case presentation

**Fig. 1:**
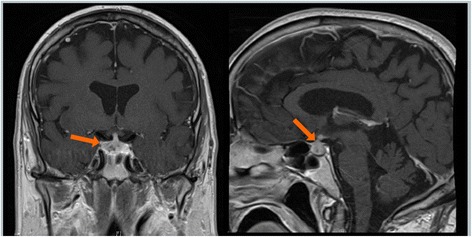
MRI brain two months prior to onset of visual complaints, demonstrating enlargement and enhancement (arrows) of the pituitary gland consistent with hypophysitis

A 53-year-old man was initially diagnosed with melanoma of the left forearm: nodular melanoma, Breslow thickness 2.4 mm, at least Clark level IV, mitotic rate of 2-4/mm^2^, no lymphovascular or perineural invasion, and no regression, ulceration, or satellitosis. He underwent wide local excision and sentinel lymph node biopsy with one lymph node with metastatic melanoma (microscopic metastases with 2 foci <1 mm) and no additional involved lymph nodes on completion lymph node dissection of the left axilla. He was followed with close observation and did not receive adjuvant therapy. The patient had consented to an institutional melanoma registry for prospective observation and data collection which was approved by the Institutional Review Board at the University of Pennsylvania. Approximately one and half years later, he presented with recurrence of his melanoma with right groin lymph node involvement and bilateral small lung metastases, *BRAF* V600 wild-type. He was treated with temozolomide and sorafenib for six cycles with initial stability of disease, then subsequent progression. During this treatment, he was diagnosed with a pulmonary embolus and received enoxaparin for anticoagulation. He was then treated with carboplatin and paclitaxel for 11 cycles. He initially tolerated treatment well and had stable disease for a period of time; however, he subsequently experienced disease progression and developed intolerable peripheral neuropathy. He then participated in a clinical trial for compassionate use of ipilimumab (prior to FDA approval) a year and a half after initial disease recurrence. He received ipilimumab at 3 mg/kg every 3 weeks for three doses. He developed a rash (Grade 2) and intermittent diarrhea (Grade 1) after his first dose of ipilimumab, both of which were managed with supportive therapy, and did not require anti-TNFalpha treatment. Nine weeks after initiation of ipilimumab, he reported new headaches. Given concern for possible hypophysitis, serum hormone levels were evaluated and found to be abnormal – cortisol −1.8 mcg/dl (6–19 mcg/dl), follicle-stimulating hormone (FSH)-16.1 mIU/ml (1.5-12.4 mIU/ml), luteinizing hormone (LH)-6.3 mIU/ml (1.7-8.6 mIU/ml), thyroid-stimulating hormone (TSH)-0.07 (0.27-4.2 mIU/ml), and testosterone-24 ng/dL (280–800 ng/dL). Magnetic resonance imaging (MRI) of the brain confirmed inflammation and edema of the pituitary gland consistent with a diagnosis of hypophysitis (Fig. 1). The fourth dose of ipilimumab was held and prednisone 1 mg/kg/day, testosterone replacement, and thyroid hormone replacement were initiated. His headaches resolved with steroid treatment.

He presented 4 months after initiation of ipilimumab with shortness of breath and acute vision loss in his left eye while on prednisone taper (40 mg daily) and therapeutic enoxaparin. Work up revealed a new small pulmonary embolus. Ophthalmological examination revealed no light perception vision in the left eye along with a left afferent pupillary defect, optic nerve swelling, and retinal whitening (Table 1). MRI of the brain and orbits, magnetic resonance angiogram (MRA) of the cerebrovascular system, carotid dopplers and an echocardiogram with bubble study were unremarkable without evidence of brain or orbital metastases. Neuro-ophthalmic evaluation revealed findings consistent with an ophthalmic artery occlusion. The vision in his left eye remained at no light perception and he continued on a steroid taper and his enoxaparin was increased to twice daily dosing.

**Table 1:** Diagnostic Tests and Workup of Patient’s Vision Loss

Time after initial ipilimumab treatment	Four months	Five months		Five and a half months	Six months	Fifteen months
Steriod Dosing/immunosuppression	Prednisone 40 mg with taper to 20 mg		Methyprednisolone IV 1 gram x 3 days, then prednisone 40 mg	Methyprednisolone IV 1 gram x 5 days, then prednisone 100 mg daily, tapered to 80 mg daily	Methylprednisolone IV 1 gram daily x 10 days, then prednisone 100 mg daily, mycophenolate mofetil 1000 mg BID	Mycophenolate mofetil 1000 mg BID, slow prednisone taper
Ophthalmologic Exam	Left Eye -No light perception Vision; Left afferent pupillary defect; Optic nerve swelling; retinal whitening	Right Eye- visual acuity 20/50; decreased color vision; visual field constriction; optic disc swelling; Left eye - vision remained no light perception	Right Eye - subjective vision improvement; reduced optic disc swelling; following steroid treatment	Right Eye- declined visual acuity		Right Eye - visual acuity 20/20; resolved optic disc swelling; Left Eye -no light perception; atrophic optic nerve
Magnetic Resonance Imaging	Unremarkable; No metastases	Unremarkable		Circumferential enhancement of intraorbital optic nerves, right > > left	Negative for brain metastases	
Lumbar Puncture						
Opening pressure				23		
Cell count		WBC 62 RBC 2	WBC 69 RBC 1	WBC 42 RBC 1		WBC 7 RBC 0
Protein (normal 15-45)		104	96	51		32
Glucose (normal 40-70)		55	52	86		112
Infectious work-up			Fungal Cx neg; AFB stain neg; Crytpo Ag neg; HSV PCR neg; RPR neg; Lyme neg; CMV neg; VZV; neg; Bartonella neg	Cx neg		Cx neg
Cytology			Negative for malignancy	Negative for malignancy		Negative for malignancy

*Abbreviations*: *IV* intravenous; *BID* twice a day, *AFB* acid fast bacili, *CMV* cytomegalovirus, *Crypto* - cryptococcus; *Cx* culture, *HSV* herpes simplex virus; neg - negative; *RPR* rapid plasma reagin (syphilis), *VZV* varicella zoster virus

**Fig. 2:**
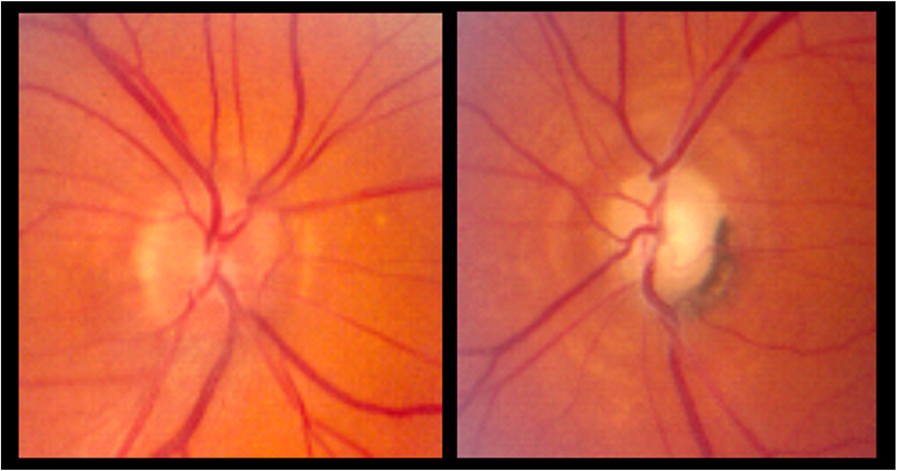
Fundus photos, taken after onset of right eye blurred vision, demonstrating mild swelling of the right optic disc (*left*) and pallor of the left optic disc (*right*)

**Fig. 3:**
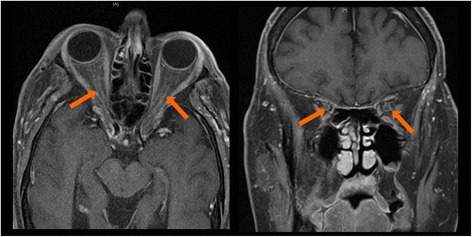
MRI orbits after onset of right eye vision loss, demonstrating subtle circumferential perineural enhancement of bilateral optic nerves, demonstrated on axial and coronal views consistent with optic nerve inflammation (highlighted by the arrows)

Five months after the initiation of ipilimumab, he described blurred vision in his right eye along with postural amaurosis. Ophthalmologic examination was notable for visual acuity of 20/50 in the right eye with associated right eye decreased color vision, visual field constriction, and optic disc swelling; left eye vision remained no light perception (Fig. 2). He was admitted to the hospital and work-up included a normal head computed tomography (CT) scan, brain MRI and magnetic resonance venography (MRV). Two lumbar punctures were performed and revealed cerebrospinal fluid (CSF) with elevated white blood cells (WBC) (lymphocytic predominance) and protein, but negative for malignancy or infection (Table 1). He was continued on enoxaparin for a possible embolic or thrombotic etiology of visual loss. The elevated CSF white blood cells and protein raised concern for an inflammatory optic neuropathy and aseptic meningitis, prompting treatment with methylprednisolone one gram intraveneously (IV) daily for three doses followed by an increased prednisone dose. The patient reported subjective improvement in his right eye vision and the optic disc swelling improved. However, three days following his last dose of methylprednisolone, the vision in his right eye worsened and he developed a headache. He was readmitted to the hospital and repeat MRI of the brain and orbits demonstrated circumferential enhancement of the right greater than left intraorbital optic nerves (highlighted by arrows) suggestive of optic neuritis (Fig. 3). MRA of the cerebrovascular system did not demonstrate any significant arterial stenoses. Repeat lumbar puncture demonstrated elevated WBCs and protein and was negative for infection or malignancy. The enhancement of the optic nerve and the noninfectious inflammatory CSF findings suggested an immune-mediated optic neuritis related to ipilimumab. He received five days of IV methylprednisolone and the vision in his right eye stabilized. He was discharged on a prednisone taper, but had a third recurrence of blurred vision in his right eye, double vision, and headaches. He was readmitted to the hospital for IV steroids and further evaluation. His vision stabilized with intravenous methylprednisolone 250 mg every six hours for 10 days. Given multiple failed attempts at steroid weaning, he was initiated on mycophenolate mofetil 1000 mg twice daily and then successfully transitioned to prednisone 100 mg daily without changes in vision. He again had no evidence of brain metastasis and stable inguinal lymphadenopathy on repeat imaging.

Fifteen months after the initiation of ipilimumab, he was clinically stable on mycophenolate mofetil and a slow prednisone taper; his ophthalmologic examination was notable for visual acuities of 20/20 in the right eye, and no light perception in the left eye; the right optic disc swelling had resolved, and the left optic nerve remained atrophic. He was again intolerant of further steroid taper with recurrent right eye blurred vision prompting hospital re-admission and another course of intravenous methylprednisolone 250 mg every 6 h. During this stay, he received five treatments with plasmapheresis. A lumbar puncture performed during this hospital stay was negative for malignancy or infection and he was discharged on mycophenolate mofetil 1000 mg twice daily and prednisone 80 mg daily (Table 1). At this time, his metastatic melanoma was slowly progressing with increasing lymphadenopathy and his visual acuity ranged from 20/20 to 20/40 in the right eye, and no light perception in the left eye. He also experienced several adverse effects from high dose steroids, including catatonic depression, severe deconditioning, and steroid myopathy.

Seventeen months after initiation of ipilimumab, the patient experienced increased fatigue, lightheadedness, and multiple falls at home. Head imaging demonstrated multiple hemorrhagic brain metastases. Anticoagulation was stopped and an inferior vena cava filter was placed. Four days later the patient was found to be obtunded. Comfort measures were initiated and the patient passed away shortly afterward.

## Discussion

With the increased use of checkpoint inhibitors, antagonist CTLA-4 and PD-1 and PDL-1 antibodies, and the growing field of immuno-oncology, the recognition and treatment of immune-related side effects is crucial. This case highlights several issues: a patient can develop multiple IrAEs, IrAEs can develop while on steroid treatment, IrAEs can be delayed in onset, steroid therapy alone may not be sufficient to effect and maintain control of IrAEs, and the treatments required for management of IrAEs (ie steroids, immunosuppressive therapy) may have significant adverse effects. Most importantly, we highlight a rare complication of optic neuropathy.

Initially, the patient’s left eye vision loss was thought to be vascular in nature, given a new pulmonary embolus, the acute onset of his vision loss, and the retinal findings of optic disc swelling and retinal whitening in the setting of ongoing treatment with high dose prednisone for previously diagnosed hypophysitis. The possibility of an ocular IrAE was discussed from the outset but the presentation was not determined to be concordant. Although it is possible that a vascular mechanism as the result of drug effect is still possible, and there are rare reports of vascular related issues, there is nothing definitive to suggest this. Shortly thereafter, the second eye then became affected in a fashion more typical of an inflammatory optic neuritis.

Importantly, these new and different visual complaints in the right eye occurred despite full anti-coagulation and prednisone therapy. The initial MRI of the brain and orbits was unrevealing and the clinical findings in the right eye were distinct from those previously identified in the left eye. However, repeat MRI imaging of the orbits revealed bilateral subtle circumferential perineural optic nerve enhancement suggestive of optic nerve inflammation. This feature, in conjunction with the noninfectious inflammatory CSF, supported the diagnosis of atypical optic neuritis from an immune-mediated process. High dose steroid therapy stabilized the right eye vision but the left eye vision never improved after initial presentation, again supporting a probable vascular cause for the left eye visual loss. This may or may not have been secondary to an inflammatory/immune-mediated process (ie, local thrombosis related to inflammation/vasculitis). Given intolerance of even small tapering of steroids, mycophenolate mofetil was selected due to its use in the treatment of other inflammatory/immune-mediated optic neuropathies as well as for its possible penetration into the CNS.

The patient’s visual loss in the right eye was not consistent with an ischemic optic neuropathy as optic nerve ischemia causes permanent visual impairment and is not steroid dependent. Infectious or parainfectious optic neuropathies, although possible in this immunosuppressed patient, were also felt to be unlikely due to an extensive laboratory work up which was unremarkable. This included negative cytomegalovirus (CMV), varicella zoster virus (VZV), rapid plasma reagin (RPR), lyme and bartonella titers. The patient never developed findings suspicious for retinal metastases or paraneoplastic retinopathy. Finally, acute demyelinating optic neuritis was considered in the differential given the enhancement of the optic nerve seen on MRI. However, during a follow up of approximately one and half years no additional or new neurological complaints developed and multiple MRI scans during this period were normal with no demyelinating lesions noted. Neuromyelitis optica (NMO) antibodies were not ordered.

It is noteworthy that the visual symptoms developed while on steroid therapy and 4 months after the last dose of CTLA-4 blockade. Given treatment with ipilimumab accompanied by the well described immune related adverse event of hypophysitis, it is reasonable to conclude that the optic nerve involvement was also an immune-related adverse event attributable to ipilimumab. Kaehler et. al. reported a characteristic pattern of immune-mediated side effects related to ipilimumab use, with some manifestations [29, 38] occurring weeks following treatment.

The immune mediated mechanism of action of these side effects is unique to checkpoint blockade and, unlike other drug-mediated side effects, is a T-lymphocyte (T cell) mediated process. Moreover, it is thought that there is loss of recognition of self-antigens and decreased self-tolerance [39, 40], which then results in an autoimmune response. Histologically, tissues affected by immune responses (both antitumor and adverse events) after ipilimumab treatment demonstrate a T-cell infiltrate [29], however multiple types of infiltrates have been observed – neutrophilic, lymphocytic, and mixed neutrophilic and lymphocytic infiltrates [26, 29]. The recommended therapy for IrAEs involves suppression of T-cell function, with steroids or other immunosuppressive agents, which interestingly does not interfere with the anti-tumor effect of ipilimumab [24]. This patient’s visual loss was severe in one eye and recurred multiple times in the other eye requiring many courses of steroids, plasmapheresis, IVIG, and mycophenylate mofetil.

## Conclusion

This patient with metastatic melanoma developed hypophysitis related to treatment with ipilimumab, and subsequently developed acute left vision loss and waxing and waning vision loss of the right eye consistent with an inflammatory optic neuritis attributed to CTLA-4 blockade. This case emphasizes a rare and refractory IrAE. The recognition of immune-related adverse events is not limited to oncologists, but extends to physicians in other subspecialties that will be involved in the care of patients receiving immune modulating therapies.

## Abbreviations

CSF: Cerebrospinal fluid
CT: Computed tomography
CTLA4: Cytotoxic T-lymphocyte antigen 4
ECT: Electroconvulsive therapy
FDA: Food and drug administration
FSH: Follicle stimulating hormone
IrAEs: Immune related adverse events
IV: Intravenous
IVC: Inferior vena cava
IVIG: Intravenous immunoglobulin
LH: Luteinizing hormone
MRA: Magnetic resonance angiogram
MRI: Magnetic resonance imaging
MRV: Magnetic resonance venography
PD1: Programmed death 1
PE: Pulmonary embolism
TNF: Tumor necrosis factor
TSH: Thyroid stimulating hormone
